# Iridochorioretinal coloboma associated with buried optic nerve drusen: a case report

**DOI:** 10.5935/0004-2749.20220037

**Published:** 2022

**Authors:** Leyre Lloreda Martin, Virtudes De la Puente Azpitarte, Hugo Santiago Balsera, Pablo Gili

**Affiliations:** 1 Department of Ophthalmology, Hospital Universitario Fundación Alcorcón, Madrid, Spain; 2 Hospital Universitario Infanta Cristina, Madrid, Spain

**Keywords:** Coloboma, Retina/abnormality, Optic nerve disease, Optic disk drusen, Tomography, optical coherence, Visual acuity, Human, Case report, Coloboma, Retina/anormalidade, Doença do nervo óptico, Drusas do disco óptico, Tomografia de coerência óptica, Acuidade visual, Humano, Relato de caso

## Abstract

Improper closure of the embryonic fissure results in ocular coloboma. Optic nerve head drusen are hyaline deposits located anterior to the lamina cribosa that grow and calcify over time. It is rarely associated with ocular coloboma, with only two cases reported. We present a patient with an irido-chorioretinal coloboma, poorly defined optic nerve limits in the right eye, and increased papillary vascular ramification and peripapillary atrophy in the left eye, without any visible drusen. Fundus autofluorescence, high-resolution optical coherence tomography, and B-scan ultrasonography confirmed the diagnosis of bilateral buried optic nerve head drusen. The association between irido-chorioretinal colobomas and optic nerve drusen in the absence of a systemic disease is exceptional. Our case demonstrates that multimodal imaging is important to correctly diagnose buried optic nerve head drusen.

## Abstract (pt)

O coloboma ocular é o resultado de um fechamento impróprio da fissura embrionária. As drusas da cabeça do nervo óptico são depósitos hialinos localizados anteriormente à lâmina cribosa que crescem e se calcificam com o tempo. A associação de ambos é rara, com apenas dois casos descritos na literatura. Apresentamos um paciente com coloboma irido-coriorretiniano e limites do nervo óptico mal definidos em seu olho direito, e aumento da ramificação vascular papilar e atrofia peripapilar em seu olho esquerdo, sem drusas visíveis. Autofluorescência de fundo, tomografia de coerência óptica de alta resolução e ultrassonografia B-scan foram realizadas confirmando o diagnóstico de drusas enterradas bilaterais da cabeça do nervo óptico. A associação entre colobomas irido-coriorretinianos com drusas do nervo óptico na ausência de doença sistêmica é excepcional. Nosso caso demonstra a importância da imagem multimodal para o diagnóstico correto de drusas enterradas de cabeça do nervo óptico.

## INTRODUCTION

Ocular coloboma is a congenital defect resulting from the improper closure of the embryonic fissure^(1)^. Patients with coloboma may be asymptomatic or exhibit leukocoria, visual acuity loss, or visual field loss^(1,2)^.

Optic nerve head drusen (ONHD) are calcified hyaline deposits located anterior to the lamina cribosa, sec ondary to an abnormal intracellular metabolism of the axons. They are frequently bilateral (69%-73%) and are classified into visible (when they protrude from the disk) and buried, which may be confused with papilledema(3,4).

Similarly, ONHD can be asymptomatic or display visual acuity reduction or visual field defects^(4)^.

Currently, the association between ONHD and iridofundal coloboma in a patient without any systemic implications has only been reported twice^(2,5)^.

## CASE REPORT

We present the case of a healthy and asymptomatic 24-year-old male who visited our clinic for routine examination. His visual acuity was 8/10 in his right eye and 10/10 in his left eye.

He had no any past medical or familial history of any ophthalmologic disease or refractive errors and no history of trauma, cardiovascular, neurologic disease, or general medical disease.

**Figure 1:**
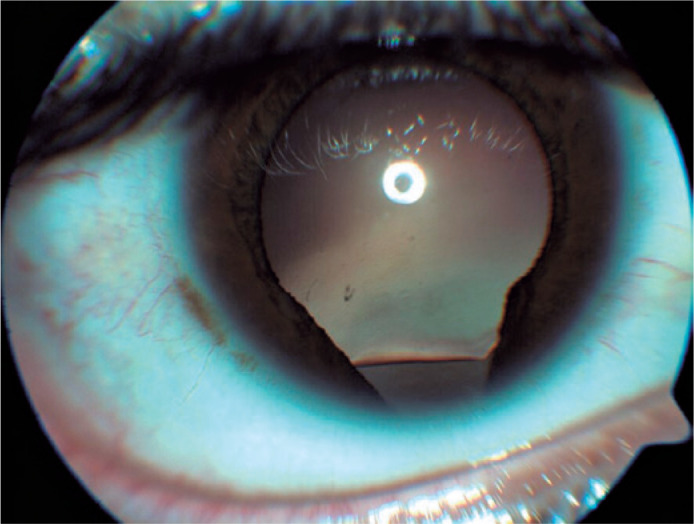
Iris and lens coloboma in the right eye.

In slit lamp examination, his right eye had an iris and lens coloboma (Figure 1), while his left eye has a normal anterior segment, with intact corneal sensation and a normal intraocular pressure (16 mmHg on both eyes).

**Figure 2:**
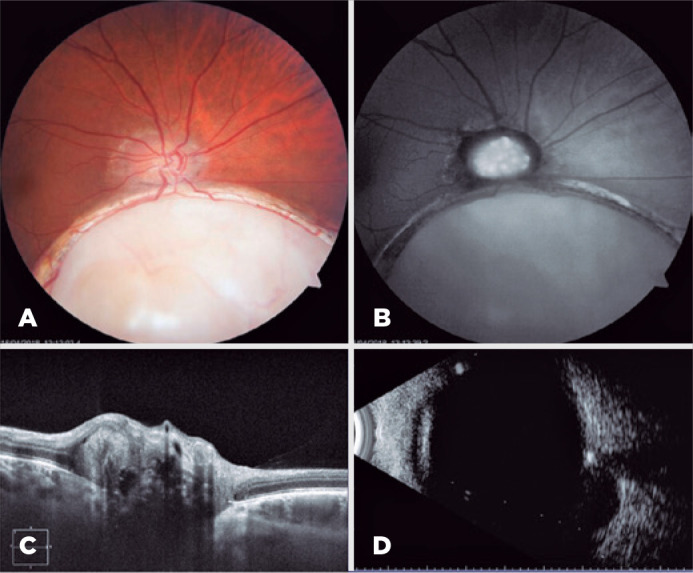
Right eye. 2A) Funduscopic examination. 2B) Fundus autofluorescence. 2C) Optic nerve OCT. 2D) B-scan ultrasonography.

**Figure 3:**
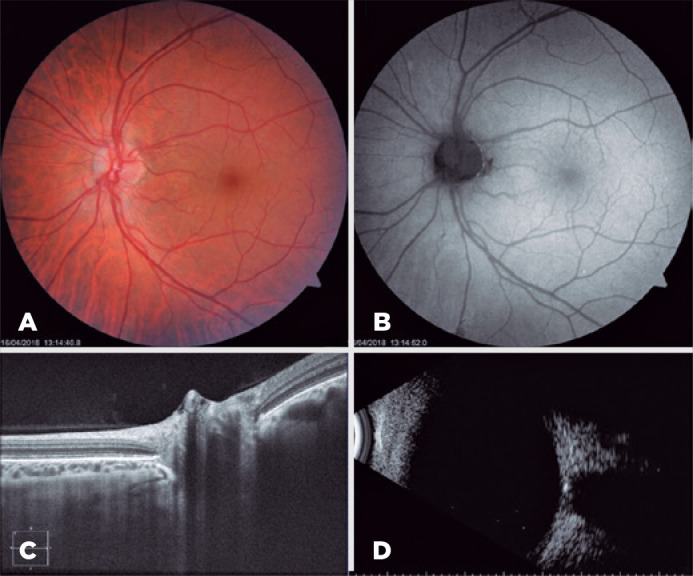
Left eye. 3A) Funduscopic examination. 3B) Fundus autofluorescence. 3C) Optic nerve OCT. 3D) B-scan ultrasonography.

Funduscopic examination revealed that his right eye had a macula-sparing chorioretinal coloboma in the inferonasal quadrant and a slightly dented optic nerve head, with diffuse elevation, peripapillary atrophy, and early papillary vessel branching (Figure 2A). Meanwhile, the left eye only showed an early branching of the papillary vessels (Figure 3A).

This young and asymptomatic patient had venous pulsations but had no hyperemia or obscuration of surface vessels. Thus, we suspected a pseudopapilloedema secondary to buried ONHD. Therefore, fundus autofluorescence (FAF), high-resolution optical coherence tomography (OCT), and ocular B-scan ultrasonography were conducted.

The FAF examination revealed that the right eye’s optic nerve had autofluorescent structures with irregular borders (Figure 2B), while that in the left eye obtained normal findings (Figure 3B).

In OCT, the right eye’s optic nerve had multiple subretinal masses with a hyporeflective core surrounded by a hyperreflective margin or border (Figure 2C); however, the findings in the left eye were unclear (Figure 3C).

B-scan ultrasonography through the optic nerve head of both eyes demonstrated a highly reflective structure with acoustic shadowing that persisted at a very low gain setting (27 dB); thus, the patient indeed had bilateral buried ONHD (Figure 2D, 3D).

The patient did not require any treatment. Instead, he is annually examined to watch for any possible complications.

## DISCUSSION

ONHD are hyaline, often calcified, deposits that are generally asymptomatic and are bilateral in 69%-73% of cases^(4,6)^.

They can be visible or buried; buried cases are not directly visible but can cause optic disk elevation with buried or obscured optic disk margins^(6,7)^.

Though ONHDs are usually asymptomatic, as in the case of our patient, they might still lead to decreased visual acuity and peripheral visual field loss^(4)^.

Therefore, buried drusen should be included in the differential diagnosis of optic disc swelling to avoid providing patients with extensive examinations for resolving increased intracranial pressure^(7,8)^.

Moreover, ONHD is often accompanied with anomalous retinal vessels with increased branching, tortuosity, and absent hyperemia^(7,8)^, which were all observed in our patient’s fundus examination but still did not lead to a definite diagnosis. Therefore, many other diagnostic methods, such as autofluorescence, B-scan echography, and OCT, were performed.

Over the last years, OCT has dramatically enhanced the ability to evaluate neuro-ophthalmic diseases^(7,8)^. This method is noninvasive and can detect optic nerve swelling, atrophy, and injury to the retinal layers. However, the depth of spectral domain-OCT (SD-OCT) imaging is limited when assessing ONHD; thus, the attention has been shifted to enhanced depth imaging-OCT (EDI-OCT)^(8)^. Using EDI-OCT, we can evaluate the entire nerve head up to the lamina cribosa, which is the most posterior extent where ONHD may be found. Therefore, between SD-OCT, EDI-OCT, and B-scan ultrasonography, EDI-OCT has the highest rate of detection of ONHD^(9)^.

Coloboma is a congenital defect of the eye caused by improper closure of the embryonic fissure^(1)^, occurring in 0.14% of the general ophthalmic population. It may involve the optic nerve alone or may be of the retinochoroidal variety^(9)^, as seen in our patient’s case. 296 Arq Bras Oftalmol. 2022;85(3):294-6

Our patient also exhibit iris and lens coloboma; thus, the defect can extend anteriorly and produce an inferonasal gap.

Congenital abnormalities of the optic disc may be associated with other congenital disorders of the eye and often, central nervous system malformations. They may also be associated with a higher risk of retinal detachment, retinoschisis, macular edema, choroidal neovascularization, and lipid exudation^(3)^.

The combination of bilateral ONHD and unilateral iris, lens, and chorioretinal coloboma in a young healthy patient is indeed a rare case. Of note, if we detect a coloboma of the optic nerve, we could be facing some syndromes, such as Walker Warburg, Aicardi, Goldenhar, Charge, or Coach syndrome. Hence, ophthalmologists need to consider these possible associations to alert pediatricians for further examinations^(1)^.

This case also demonstrates that when a patient exhibits pseudopapilloedema, using multiple imaging methods, such as ultrasonography, autofluorescence and OCT, is important to correctly diagnose buried ONHD, thereby preventing unnecessary invasive and expensive diagnostic tests, including lumbar puncture and magnetic resonance imaging.
